# Voice outcome after unilateral ELS type III or bilateral type II resections for T1‐T2 glottic carcinoma: Results after 1 year

**DOI:** 10.1002/hed.25582

**Published:** 2019-01-16

**Authors:** Yda van Loon, Martine Hendriksma, Bas J. Heijnen, Vivienne A. H. van de Kamp, Marieke M. Hakkesteegt, Stefan Böhringer, Ton P. M. Langeveld, M. A. de Jong, W. Martin C. Klop, Robert J. Baatenburg de Jong, Elisabeth V. Sjögren

**Affiliations:** ^1^ Department of Otorhinolaryngology, Head & Neck Surgery Leiden University Medical Center Leiden The Netherlands; ^2^ Department of Otorhinolaryngology, Head & Neck Surgery Erasmus MC Cancer Institute Rotterdam The Netherlands; ^3^ Department of Biomedical Data Sciences Leiden University Medical Center Leiden The Netherlands; ^4^ Department of Radiotherapy Leiden University Medical Center Leiden The Netherlands; ^5^ Department of Head and Neck Surgery Netherlands Cancer Institute/Antoni van Leeuwenhoek Amsterdam The Netherlands

**Keywords:** anterior commissure involvement, early glottic cancer, laser surgery, TLM, voice outcome

## Abstract

**Background:**

Voice outcome was assessed in patients with extended T1 and limited T2 glottic carcinoma, treated with a unilateral type III or a bilateral type II resection according to the European Laryngological Society (ELS) classification.

**Methods:**

Objective evaluation (acoustic and aerodynamic parameters), perceptual evaluation (GRBAS), and patients' self‐assessment (voice handicap index [VHI]) were performed before and 1 year after treatment. Results were evaluated according to ELS resection type and the involvement of the anterior commissure.

**Results:**

The majority of voice parameters in all resection subgroups showed an improvement of the mean score 1 year postoperatively. Grade of dysphonia varied between 1.15 and 1.66 postoperatively and VHI score varied from 23.3 to 24.5.

**Conclusion:**

Voice outcome after ELS unilateral type III or a bilateral type II resection for extended T1 and limited T2 glottic carcinoma is good with mild to very moderate perceptive dysphonia and low self‐reported voice impairment.

## 1 INTRODUCTION

The two main treatment modalities for early glottic carcinoma (Tis‐T2) are transoral CO_2_ laser microsurgery (TLM) and radiotherapy (RT). There is no randomized study that proves that one treatment modality is more effective than the other and both are still widely used.^1^ However, several studies indicate that although both treatments show similar local tumor control, TLM as a primary treatment modality is associated with a higher laryngeal preservation rate than RT^2^, ^3^, ^4^, ^5^ as TLM leaves all treatment options open in the case of recurrent disease.

In addition to oncological outcome, functional outcome—mainly voice outcome—is an important factor when choosing a primary treatment for these patients. It has been shown that the postoperative voice outcome in limited resections such as subepithelial and subligamental resections (type I‐II according to the European Laryngological Society [ELS] classification^6^) is normal or near to normal^7^, ^8^ and that voice outcome for TLM and RT in these lesions is comparable.^2^, ^7^, ^9^, ^10^, ^11^ However, the postoperative voice is generally poorer in more extended resections (type III‐IV) than in superficial resections (type I‐II).^12^, ^13^, ^14^, ^15^, ^16^, ^17^, ^18^ This is consistent with greater glottal insufficiency due to tissue loss.^12^, ^13^ Moreover, when the tumor extends to the anterior commissure (AC), poorer voice outcome is seen, regardless of the treatment modality.^16^, ^19^, ^20^ When treated with TLM, the best voice results are achieved when the AC can be left intact along with part of the vocal fold muscle.^21^

Although the use of TLM is expanding, there is relatively little data investigating voice outcome after more extensive unilateral resections (type III‐VI) or bilateral resections and no data on the comparative outcome for RT in these larger lesions. This is one of the reasons that the “Dutch guideline for the Treatment of Laryngeal Carcinoma” considers TLM as the preferred treatment only for superficial T1a midcord lesions, which are resectable with a subepithelial or subligamental resection (type I or II), with RT being the recommended treatment in more extended T1 and T2 glottic lesions.^22^

Therefore, the aim of this study was to investigate voice outcome in patients with early glottic carcinoma with an extended T1 or limited T2 lesion requiring a unilateral transmuscular resection or a bilateral subligamental resection. If functionally acceptable results can be achieved in our setting, these resections can then be considered for routine treatment of extended T1 and limited T2 glottic carcinoma. This would be an addition to the unilateral subepithelial and subligamental resections already performed in the Netherlands, giving these patients the benefit of additional treatment options in the case of a recurrence.

## 2 MATERIAL AND METHODS

### 2.1 Patients

From December 2009 to March 2015, a non‐randomized multicenter prospective outcome study on functional outcome after treatment for early glottic carcinoma (extended T1N0 and limited T2N0) was started in three Dutch tertiary referral hospitals (University Cancer Center Leiden ‐ The Hague, Erasmus Medical Center Cancer Institute, and Netherlands Cancer Institute/Antoni van Leeuwenhoek). The target population of the study was patients with extended T1N0 and limited T2N0 glottic tumors that if treated surgically would require a unilateral transmuscular resection—defined as a type III cordectomy according to the ELS classification or a bilateral subligamental resection which is does not have an official ELS classification but would be best described as a bilateral type II. To clarify these bilateral type II resections, they consisted of subligamental resections on both sides of the vocal cord including the AC, leaving the muscle and its anterior attachment to the perichondrium intact. Some of the resections were staged to prevent web formation. Preliminary inclusion in the study took place in the outpatient clinics. All patients with a lesion possibly fitting the above criteria were offered the choice between TLM or RT after counseling, which was supplemented by written information on the study treatments.^23^ Definite inclusion in the study occurred during endoscopy for patients confirmed at that time to have lesions fitting the inclusion criteria. Patients were then treated according to their preference. Of the eligible patients, only four (6.3%) chose RT. Due to the differences in group size between TLM and RT, contrary to initial study plans, patients who were treated with RT were not included in this study. Total and extended cordectomies (type IV‐VI) were also excluded as they are currently viewed as too aggressive for functional reasons for primary treatment of early glottic carcinoma in the Netherlands and are therefore not performed on a routine basis. Other exclusion criteria were the inability to speak and read the Dutch language, preexistent problems with the voice or swallowing, previous radiation for head and neck tumor(s), and the presence of any psychological, familial, sociological, or geographical condition potentially hampering compliance with the study protocol or follow‐up schedule. Also patients who had recurrent disease in the first year after treatment were excluded because the recurrence and the subsequent treatment were of potential influence on the functional outcomes. In the study protocol, in case of positive margins a second surgery could be carried out, and in case of bilateral spread, the procedure could be staged. Voice outcome of patients who participated in the study was investigated before treatment and 1 year after treatment. During both visits, speech was recorded and a self‐administrated questionnaire, as described below, was filled in. The study was approved by the local medical ethics committees at all three hospitals and written informed consent was obtained from all patients before inclusion in the study.

### 2.2 Subjective evaluation

#### 2.2.1 Perceptual voice evaluation

The perceptual analysis was performed on a running speech voice sample, using the GRBAS‐rating scale.^24^ This rating consists of five domains: grade (G), roughness (R), breathiness (B), asthenia (A), and strain (S). Only the grade (henceforth G‐score) was rated. A panel of five experienced listeners (E.V.S., V.A.H. vdK., B.J.H., M.M.H, and Y.vL.) familiar with the GRBAS‐system, who were blinded to all data, conducted the voice evaluation. Speech material for the perceptual analysis consisted of a standard phonetically balanced Dutch text “80 dappere fietsers” (80 brave cyclists). Speech samples, with an average duration of 30 seconds, were presented in a random sequence, before or after treatment, and all samples were rated by consensus. If the listeners scored the sample differently, consensus was reached through reevaluation and discussion. The outcome of each sample was classified from 0 to 3 (0 = normal; 1 = mild; 2 = moderate; and 3 = severe): a higher score represents a more dysphonic voice.^24^

#### 2.2.2 Patient‐self assessment

A patient self‐report on the voice impact was completed by the patients during their visits to the outpatient clinic by scoring the voice handicap index (VHI).^25^ The Dutch version of the VHI is a validated questionnaire measuring the voice impairment in daily life.^26^ The questionnaire comprises 30 questions on a 5‐point Likert scale, with grading from 0 to 4 (0 = never and 4 = always). A total of 15 points or more out of a possible 120 points is taken to indicate voice impairment in daily life.^27^ A higher score represents a worse voice handicap. A clinically relevant change is defined as a 10‐point shift for individuals and as a 15‐point shift for groups.^28^ The VHI scores were also categorized into five different groups: a score of 0 (normal voice), scores of 1 to 30 (slight voice impairment), scores of 31 to 60 (moderate voice impairment), scores of 61 to 90 (severe voice impairment), and scores from 91 to 120 (very severe voice impairment).^13^

### 2.3 Objective evaluation

Recordings were made at a sampling frequency of 44.1 kHz with a dual microphone headset recorder from Alphatron Medical Systems recorder and with a Beyer dynamic microphone, type Opus 56, Germany, in a noise‐free environment. Intensity and frequency measurements in a phonetogram were obtained with an automatically recording Voice Profiler (VRP) (2007, Alphatron, Rotterdam, the Netherlands). Acoustic parameters (mean speaking fundamental frequency in Hertz [Hz] and range Sound Pressure Level [SPL] in decibel [dB]) were analyzed using the program VRP. The aerodynamic parameter (s/z ratio) was analyzed using the program Audacity (Boston, Massachusetts). For the s/z ratio, the longest waveform of each patient was recorded as their /s/ or /z/. The duration of the /s/ sound was divided by the duration of the /z/ sound, resulting in the s/z ratio.^29^ All patients were instructed in vocal hygiene directly after surgery. If patients asked for guidance or speech therapy was indicated, they were referred to a speech‐language pathologist; however, this was not included in the protocol and not standardized. Patients did not receive any speech therapy before 3 months after TLM.

### 2.4 Statistical methods

All statistical analyses were performed with SPSS version 23.0 (IBM Corp, Armonk, New York) and R version 3.4.0 (Foundation for Statistical Computing, Vienna, Austria). The level of significance was set at *P* < .05. Normal distribution assumptions were checked with the Kolmogorov‐Smirnov test and by computing skewness and kurtosis. Outcome variables in this study were G‐score, VHI, fundamental frequency, range SPL, and s/z ratio.

The effect of time on outcomes variables was assessed with a paired *t* test to compare preoperative and postoperative data in each patient. Additionally, we performed a linear mixed model analysis with a random intercept for each patient while adjusting for possible confounders: age, sex, and T classification. The preoperative and postoperative results between the subgroups on all outcome variables (unilateral type III vs bilateral type II and unilateral/bilateral resections with/without involvement of the AC) were also compared with an independent *t* test or with one‐way analysis of variance.

To account for missing data, multiple imputation analyses were performed using package *mice* in *R*.^30^ Missingness varied between 1% (preoperative s/z ratio) and 27% (fundamental frequency) and therefore 30 imputed data sets were generated. Imputation variables were all outcome variables and covariates used in the mixed models. Sensitivity analysis was carried out by comparison with the complete case analysis. Results from the individual imputed data sets were summarized by an automatic SPSS feature, relying on Rubin's rule.

All models were evaluated in two different subgroups. First, according to resection type (unilateral type III or bilateral type II resections) and second according to resection involvement of the AC (no involvement, unilateral involvement, or bilateral involvement).

## 3 RESULTS

### 3.1 Patients

**Figure 1:**
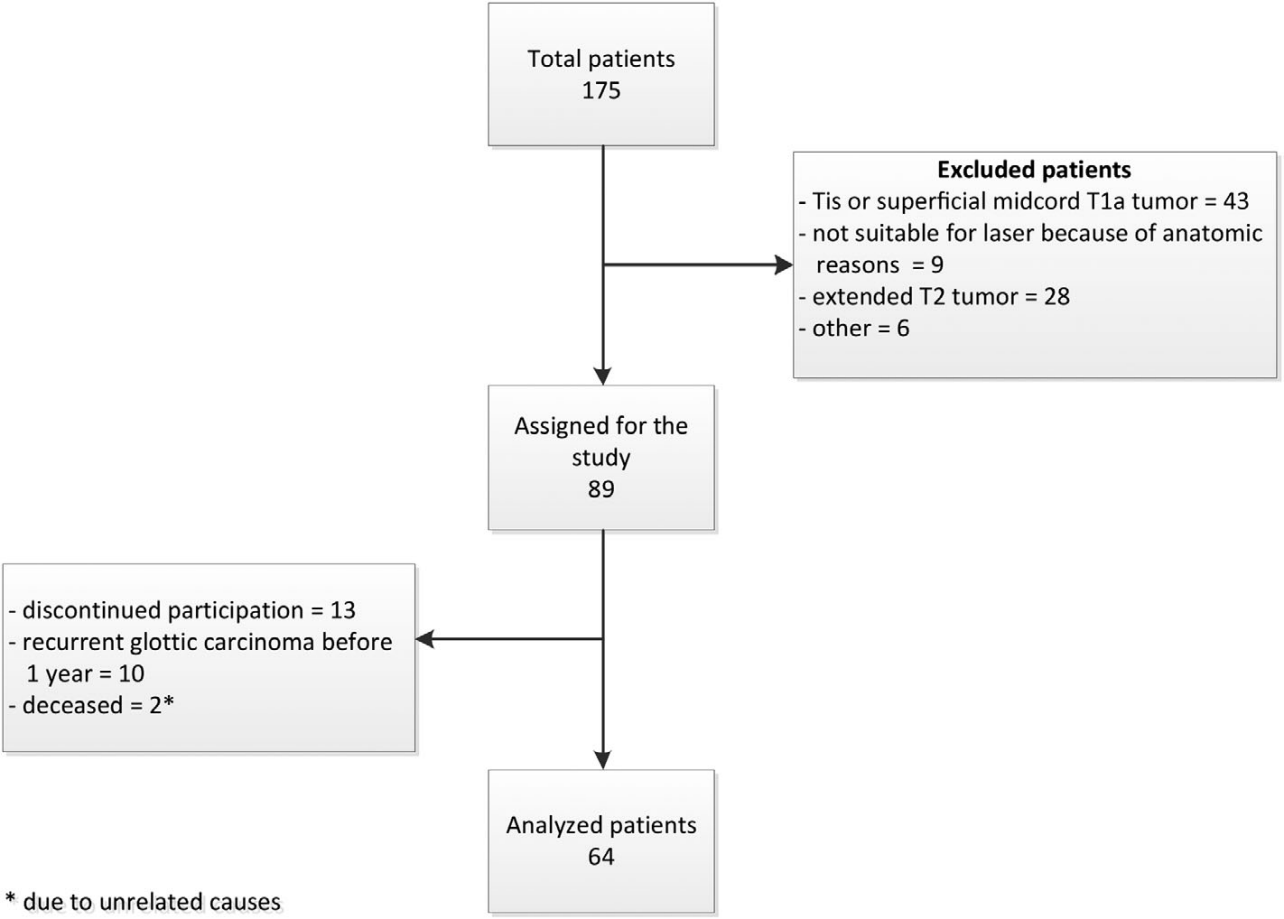
Flow diagram

In total 175 patients with suspected or proven extended T1 and limited T2 glottic carcinoma were identified as possible candidates for the study between December 2009 and March 2015. Of these, 89 patients fitted the inclusion criteria during endoscopy. Of these, 13 patients were lost‐to‐follow‐up or discontinued participation in the first 3 months of the study. Two patients died due to unrelated causes (1 patient died of urinary bladder cancer and 1 of cardiovascular disease) and 10 patients had a recurrent glottic carcinoma before 1 year of follow‐up was completed and were therefore excluded, leaving 64 patients for analysis (Figure 1).

Of the five outcome measures (G‐score, VHI, fundamental frequency, range SPL, and s/z ratio) 17.2%‐26.6% were missing. Therefore, we performed multiple imputations. Sensitivity analysis showed that our results were robust (see statistical methods). For example, the regression coefficients for complete cases and multiple imputation analysis for the VHI and s/z ratio respectively were comparable (β VHI 25.8 [SE 4.9] and 25.8 [SE 8.0]; β s/z ratio 1.24 [SE 0.19] and 1.27 [SE 0.19]).

Table 1 shows the baseline characteristics of 64 analyzed patients. In the overall group, outcomes were not significantly affected by the variables age, sex, and T classification and these were therefore not considered in further analyses. Table 2 shows voice outcome according to ELS‐resection type and Table 3 shows the results according to the resection involvement of the AC.

**Table 1:** Baseline characteristics

Characteristics	No. of patients (%) Total = 64 (100%)
Mean age at surgery (SD)	67.4 (9.2)
Sex	
Male	54 (84.4)
Female	10 (15.6)
T classification	
T1a	29 (45.3)
T1b	20 (31.3)
T2	15 (23.4)
ELS resection	
Type III	40 (62.5)
Type II bilateral	24 (37.5)
AC involvement	
No	13 (20.3)
Unilateral	27 (42.2)
Bilateral	24 (37.5)

Abbreviations: AC, anterior commissure; ELS, European Laryngological Society; T, tumor.

**Table 2:** Voice outcome according the type of resection

	Preoperative Mean (SD)	1‐year post‐TLM Mean (SD)	Mean difference (95% CI)	*P*‐value
ELS type III (no. of patients = 40)				
s/z ratio	1.13 (0.38)	1.26 (0.48)	−0.13 (−0.31; 0.04)	.13
F_0_ (Hz) male (34)	140 (29)	142 (29)	−2 (−14; 10)	.72
F_0_ (Hz) female (6)	170 (51)	187 (55)	−17 (−39; 5)	.12
Range SPL (dB)	36.7 (13.3)	38.0 (9.67)	−1.32 (−4.93; 2.30)	.47
VHI	31.8 (17.1)	23.3 (15.3)	8.47 (2.59; 14.34)	.005*
G	1.49 (0.77)	1.15 (0.70)	0.35 (0.04; 0.67)	.02*
Bilateral type II (no. of patients = 24)				
s/z ratio	1.20 (0.48)	0.98 (0.38)	0.22 (0.06; 0.38)	.009*
F_0_ (Hz) male (20)	143 (45)	158 (42)	−15 (−31; 1)	.06
F_0_ (Hz) female (4)	178 (45)	183 (42)	−5 (−40; 30)	.78
Range SPL (dB)	30.5 (13.7)	37.1 (7.4)	−6.60 (−11.16; −2.05)	.005*
VHI	28.2 (19.8)	24.5 (21.9)	3.70 (−6.23; 13.63)	.46
G	1.66 (0.88)	1.55 (0.85)	0.10 (−0.27; 0.48)	.59

Abbreviations: dB, decibel; ELS, European Laryngological Society; F_0_, mean speaking fundamental frequency; G, grade from the GRBAS‐rating scale; Hz, Hertz; range SPL, range sound pressure level; TLM, transoral CO_2_ laser microsurgery; VHI, voice handicap index.

* Statistically significant (*P* < .05).

**Table 3:** Voice outcome according to the resection involvement of the anterior commissure

	Preoperative Mean (SD)	1‐year post‐TLM Mean (SD)	Mean difference (95% CI)	*P*‐value
Without AC involvement (no. of patients = 13)				
s/z ratio	1.17 (0.38)	1.39 (0.51)	0.22 (−0.47; 0.03)	.08
F_0_ (Hz) male (10)	155 (39)	136 (33)	19 (−8; 46)	.16
F_0_ (Hz) female (3)	174 (19)	191 (28)	−17 (−49; 15)	.29
Range SPL (dB)	35.0 (16.7)	38.6 (10.1)	−3.55 (−10.7; 3.60)	.33
VHI	33.9 (18.3)	22.3 (14.9)	11.6 (2.15; 21.1)	.01*
G	1.68 (0.77)	1.13 (0.58)	0.56 (0.03; 1.08)	.4*
With AC involvement unilateral (no. of patients = 27)				
s/z ratio	1.12 (0.37)	1.21 (0.44)	−0.09 (−0.31; 0.13)	.43
F_0_ (Hz) male (23)	131 (20)	144 (28)	−13 (−23; −2)	.02*
F_0_ (Hz) female (4)	172 (64)	191 (68)	−19 (−41; 1)	.06
Range SPL (dB)	37.0 (11.4)	38.1 (9.6)	−1.01 (−4.95; 2.92)	.61
VHI	30.4 (17.0)	24.1 (15.3)	6.28 (−1.08; 13.6)	.09
G	1.41 (0.76)	1.15 (0.75)	0.26 (−0.14; 0.65)	.20
With AC involvement bilateral (no. of patients = 24)				
s/z ratio	1.19 (0.48)	0.98 (0.38)	0.22 (0.05; 0.38)	.01*
F_0_ (Hz) male (21)	145 (45)	158 (41)	−13 (−29; 3)	.10
F_0_ (Hz) female (3)	175 (20)	172 (32)	3 (−41; 46)	.90
Range SPL (dB)	31.0 (14.2)	36.7 (7.40)	−5.73 (−10.7; −0.78)	.02*
VHI	28.7 (19.6)	24.2 (22.2)	4.45 (−5.65; 14.6)	.38
G	1.66 (0.88)	1.55 (0.85)	0.10 (−0.27; 0.48)	.59

Abbreviations: AC, anterior commissure; dB, decibel; ELS, European Laryngological Society; F_0_, mean speaking fundamental frequency; G, grade from the GRBAS‐rating scale; Hz, Hertz; range SPL, range sound pressure level; TLM, transoral CO_2_ laser microsurgery; VHI, voice handicap index.

* Statistically significant (*P* < .05).

The 1‐year overall recurrence rate was 11.2% (10 out of 89 patients, T1 = 4, T2 = 6). All recurrences occurred in the unilateral type III resection group (eight patients with and two without AC involvement).

### 3.2 Subjective evaluation

#### 3.2.1 Perceptual evaluation

**Figure 2:**
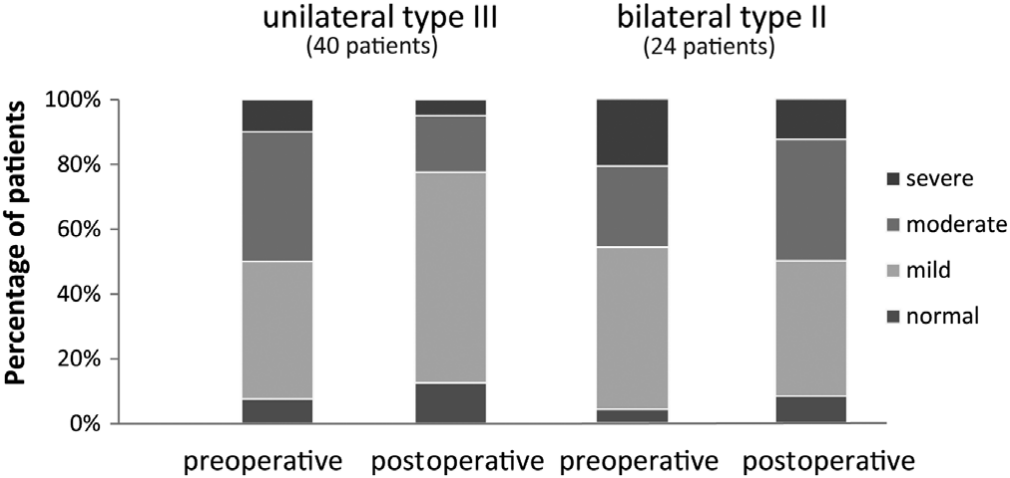
Distribution of the grade according to the type of resection

The G‐score improved in all study groups with mean postoperative G‐scores varying from 1.49 to 1.15 (unilateral type III) and from 1.66 to 1.55 (bilateral type II), corresponding to mild to moderate dysphonia (Table 2). These postoperative G‐scores were significantly lower in patients treated with type III resections compared to bilateral type II resection. There was no difference in postoperative scores in the subcategories regarding the AC involvement (Table 3). Looking at the preoperative to postoperative decrease in G‐score, this was significant in patients treated with a unilateral type III resection and resections without AC involvement. In total, more patients presented with a normal or mildly dysphonic voice postoperatively (67.2%) than preoperatively (51.6%) (Figure 2) although this difference was not significant. Conversely, 7.8% of patients were rated as severely dysphonic after surgery.

#### 3.2.2 Patient self‐assessment

**Figure 3:**
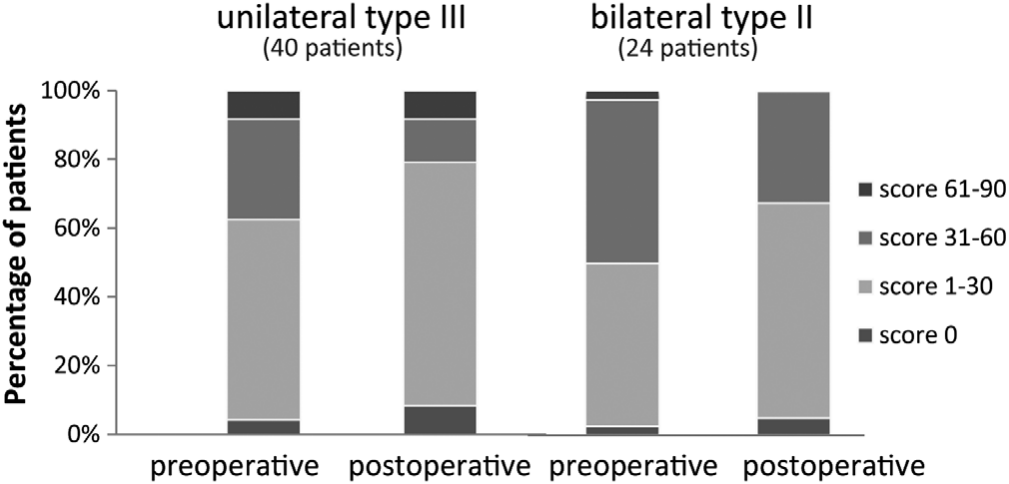
Distribution of the voice handicap index (VHI) according to the type of resection

The mean VHI scores improved in all study groups postoperatively. For the unilateral type III resections, the mean dropped from 31.8 to 23.3 points. For the bilateral type II resections, VHI scores decreased from 28.2 to 24.5 points (Table 2). The improvement in the VHI score was significant in unilateral type III resections and in resections not involving the AC (Tables 2 and 3). Looking at individual patients, 23 (35.9%) had a clinically relevant improvement of 10 points or more and 13 (20.3%) had a clinically relevant deterioration of 10 points or more. This ratio of improvement/deterioration was similar in the five subgroups (unilateral type III, bilateral type II, without AC involvement, or with unilateral/bilateral involvement). Figure 3 shows the categorized VHI scores. Postoperatively, more patients (70.3%, *n* = 45) experienced less impairment of their voice than preoperatively (54.7%, *n* = 35). This difference was not statistically significant.

### 3.3 Objective voice evaluation

#### 3.3.1 Acoustic evaluation

Range SPL increased in all study groups after treatment with scores varying from 30.4 to 38.6 dB (Tables 2 and 3). The increase in range SPL was significant in patients treated with bilateral type II (from 30.5 to 37.1 dB) and resections with bilateral involvement of the AC (from 31.0 to 36.7 dB) (Tables 2 and 3). However, there was no statistical difference between the various groups in the postoperative scores. The mean speaking fundamental frequency increased for most patients except for male patients with resections without involvement of the AC and female patients with resections with bilateral involvement of the AC. The average postoperative mean speaking fundamental frequency in males ranged from 131 Hz (resections with unilateral AC involvement) to 155 Hz (resections without involvement of the AC) and from 170 Hz (unilateral type III) to 178 Hz (bilateral type II) in females. The increase in mean speaking fundamental frequency was significant only in unilateral resections with involvement of the AC in male patients (*P* = .02) (Table 3).

#### 3.3.2 Aerodynamic evaluation

In the aerodynamic parameter (s/z ratio) we found varying changes. In some subgroups, the ratio changed toward the ideal of 1 and in other sub groups, it changed away from this value. Postoperatively, the s/z ratio was 0.98 compared to 1.2 preoperatively (bilateral type II resections and resections with bilateral AC involvement) and from 1.39 postoperatively compared to 1.17 preoperatively (resections without AC involvement) (Tables 2 and 3). The s/z ratio showed significant improvement in patients treated with bilateral type II resections and bilateral resections of the AC (Tables 2 and 3). Also, postoperative results of patients treated with bilateral type II resections were significantly better than patients treated with unilateral type III resections. Results of patients treated with bilateral resections of the AC were significantly better than patients treated without or with a unilateral resection of the AC.

## 4 DISCUSSION

In this study, we investigated voice outcome 1 year after treatment in patients with early glottic carcinoma (extended T1 and limited T2) treated with a unilateral transmuscular (type III) or bilateral subligamental (bilateral type II) resection. We also examined the results according to the involvement of the AC in the resection. For all resection subgroups, most voice parameters show an improvement of the mean score at 1 year postoperatively. This means that the defect and fibrosis from surgery are on average less of a problem for patients than the tumor itself. As for the postoperative outcome, perceptive dysphonia is mild but is statistically less after a unilateral type III resection (mild) than after a bilateral type II resection (mild to moderate). The voice handicap is universally low and similar for all treatment groups, not exceeding a mean of 24.5 out of a possible 120 points. The dynamic range of patient's voices is similar for all resection types in which values can be considered to be in the low normal to slightly decreased range depending on the reference used for normal values.^31^, ^32^ The mean speaking fundamental frequency after treatment increases and is higher than normal for all resection types in male patients but within normal values for female patients.^33^ Finally, the effect on aerodynamic efficiency of voicing varies. In our series, according to the s/z ratio, voicing is ultimately more efficient in patients treated with bilateral type II resections and bilateral resections of the AC than in patients treated with a unilateral type III resection with or without a unilateral resection of the AC. It is not clear if this change was also clinically relevant to the patients as it does not translate into less perceptive dysphonia, larger dynamic range, or lower voice handicap in these patients. Although mean values for almost all parameters improve, it is important to realize that this is for group averages. This means that there is still a group of patients that experience a decline in voice outcome scores. Although not always statistically significant, this tends to be male patients with T1b tumors including the AC.

Interestingly, for the subjective parameters (G‐score and VHI), the improvements that were statistically significant tended to be in the unilateral type III resections and resections not involving the AC, whereas statistically significant improvements in the objective parameters, if present, were found in the bilateral type II resections and resections involving the AC. This leads us to conclude—as has been done previously in literature—that these parameters all highlight different aspects of voice quality and function and that improvement in one parameter does not necessarily lead to improvement in another.^34^, ^35^

In this study, voice outcome of resections involving the AC (unilateral or bilateral) did not differ significantly from that of resections without involvement of the AC in any of the evaluated parameters, except for the s/z ratio. The s/z ratio was significantly different (and closer to 1) for patients having undergone a bilateral resection of the AC versus patients with resections with no AC involvement (s/z ratio 0.98 vs 1.39). Therefore, we do not consider involvement of the AC as a negative factor in this study and our results show acceptable voice outcome for these resections with mild to moderate dysphonia and a low VHI. However, it must be emphasized that the resections involving the AC were of limited extent and depth. The bilateral resections were subligamental and 58.3% (*n* = 14) were performed staged in time. Unfortunately, to the best of our knowledge, there is no specific literature on functional outcome of superficial bilateral resections and therefore comparing our results with previous findings was not possible. We would therefore advocate more publications on data of superficial bilateral resections.

Our recurrence rate at 1‐year was 11.2%. This cannot be compared directly to recurrence rates reported by other authors as the mean follow‐up time in most studies is longer than in our study. However, as we expect most of the recurrences to occur within the first year and as our outcome at this point compares favorably to local control rates reported for T1 (85%‐87%)^21^ and T2a (80%)^36^ in large series, we anticipate that our eventual results will be in line with literature even though additional recurrences in the following years of follow‐up are possible. It is important to acknowledge that TLM for T1 and limited T2 glottic carcinoma is based on the concept of narrow‐margin surgery and that there is ultimately a trade‐off between achieving a radical resection either during the primary resection or by means of re‐resection of positive margins and optimizing functional results. There is as of yet no definite recommendation regarding what constitutes a safe margin. Recommended resection margins vary between 0.5 and 2 mm^21^ and both wait‐and‐see and re‐resection regimes are advocated in literature.^37^

### 4.1 Review literature

The findings on functional outcomes in literature in relation to our own results are discussed per parameter below.

#### 4.1.1 Perceptual evaluation

The G‐score improved in all subgroups with postoperative scores varying from light to very moderate dysphonia. The improvement was significant in patients treated with unilateral type III resections and patients treated without a resection of the AC. In total, 70.4% of the voices in our study were rated as a normal or mildly dysphonic after treatment. Vilaseca et al^17^ found that after type II and III resections, 55% of their patients had a normal or mildly dysphonic voice. In line with these results, a study by Czecior et al^38^ found that in 33 patients, 54% had a normal or mildly dysphonic voice after type III resection .

Regarding the ELS‐resection types, the postoperative G‐score was significantly better in patients with unilateral type III resections than in patients with bilateral type II resections although there is no generally accepted value for what makes up a clinically relevant difference in G‐score. Only a few studies have presented results specifically for patients treated with a unilateral type III resection. The study by Ledda et al^14^ analyzed vocal outcomes of 141 patients with early glottic cancer after TLM. Thirty of them had a unilateral type III resection with an overall Grade of 1.29.^14^ The study by Peretti et al assessed vocal outcomes in relation to the types of endoscopic cordectomy. In their study, 11 patients with early glottic cancer had a type III resection and scored a G‐score of 1.4.^13^ Our results show a slightly lower G‐score of 1.15 postoperative for patients treated with type III resections. To the best of our knowledge, no other studies described voice outcome results for bilateral type II resections.

Regarding the AC involvement, the postoperative G‐scores for resections without involvement of the AC were very similar to those for resections with one‐sided involvement of the AC. The G‐score for resections with bilateral involvement of the AC was higher although the difference between the three was not statistically significant. This is in line with our clinical observations that one‐sided resections of the AC have relatively little additional impact on dysphonia compared to resections not involving the AC. In contrast, most studies show that resections involving the AC result in worse voice outcome with regard to the grade of dysphonia than resection without the AC.^16^, ^20^, ^39^ This may be caused by the formation of an anterior glottic scar web in some cases, but also by an increased glottal gap.^7^, ^40^ An explanation for this result could be the fact that the lesions, and therefore the depth of the bilateral resections in our study, were limited as well as the fact that the resections were staged in 14 patients. This has less effect on the glottic closure or the development of anterior glottic scars than in deeper bilateral (type V‐VI) resections.

#### 4.1.2 Patients self‐assessment

In line with the G‐score, the VHI also improved with a mean of 3.7‐11.6 points in all subgroups with postoperative scores varying between just 23.3 and 24.5 points. Again, the improvement was significant in patients treated with unilateral type III resections and patients treated without a resection of the AC. Looking at individual patients, we found the improvement to be 10 points or more in 23 patients (35.9%). Conversely, in 13 patients (20.3%), we saw a deterioration of 10 points or more. Van Gogh et al defined a clinical relevant difference score to be 10 points in individual patients and 15 points or more when comparing groups of patients.^28^ Therefore, we conclude that one‐third of patients can expect a clinically relevant improvement in their voice handicap, a little less than half can expect a similar voice handicap and a minority of one‐fifth will report a clinically relevant deterioration in their voice handicap 1 year postoperatively. These proportions were similar in the various resection subtypes.

Regarding the ELS‐resection type subgroups, the postoperative VHI scores were very similar in both subgroups. Literature has already shown that unilateral superficial type I‐II resection can provide near to normal VHI scores ranging from 9.0 to 20.7 points.^13^, ^16^, ^18^, ^35^, ^41^, ^42^, ^43^, ^44^, ^45^, ^46^ However, the depth of muscle resection in a type III resection varies according to the depth of the tumor, and in the case of deeper excisions a type III resection may induce lasting dysphonia.^7^ Only a few studies have investigated the VHI results in unilateral type III resections specifically as opposed to combining them with other resection types. Nunez Batalla et al^46^ investigated the VHI results in 19 patients with T1a tumors after a unilateral type III resection and found a mean VHI score of 28.8 points.^46^ However, their study design was cross‐sectional, meaning that patients were not all assessed at the same time‐point postsurgery although there was a minimal follow‐up time of 6 months. Therefore, comparison with our study is difficult. Fink et al^18^ assessed 12 patients with early glottic carcinoma and found an improvement of 16.6 points with a mean VHI score of 27.8 (SD 20.4) points after a median of 7 months. The difference of 16.6 points in this case could be classified as clinically relevant. For our unilateral type III resections, we found a postoperative VHI of 23.3. This is slightly lower than in the studies above. This is in line with the slightly lower G‐scores compared to literature that we found in the perceptual analysis. Both may be explained by a statistical variation due to the larger size of our cohort or by a different case‐mix with regard to the depth of the type III resection.

Regarding the AC involvement, again the VHI score was very similar in the three subgroups. In literature, as with the dysphonia grade score, involvement of the AC is generally associated with poorer voice outcomes in the VHI,^47^ although the number of studies investigating postoperative outcomes with VHI is limited.^20^ The study by Taylor et al investigated functional outcomes of T1b tumors after TLM with the VHI‐10 and without describing the ELS classification of the resections.^47^ Therefore, these results are not directly comparable with our study. The study by Lee et al compared voice outcomes according to the extent of surgery (type I‐V) and made a distinction between AC involvement or no involvement. Nineteen patients with involvement of the AC or who had bilateral vocal cord involvement revealed a tendency to or had deteriorated voice quality postoperatively. These VHI scores (47.70 SD 14.90) are much higher than in our study. Although they combined the different resection types (type I‐V) in analysis, it is probable that the majority of these resections were deeper than in our series.^41^ To the best of our knowledge, no study has presented voice outcome specifically for AC involvement in either unilateral type III or bilateral type II resections.

#### 4.1.3 Objective evaluation

##### Acoustic parameters

The range SPL improved in all subgroups with values that can be considered within the normal range.^32^ The improvement was significant in patients with a bilateral type II resection and in patients with bilateral resections of the AC. The fact that all patients showed a trend toward postoperative improvement of the range SPL means that they had a wider range between the softest and loudest voicing after treatment. Functionally, this leads to more dynamic possibilities resulting in a larger range in loudness of speech. In line with our results, the study by Jotic et al showed a significant improvement of range SPL for 19 patients with early glottic carcinoma (Tis‐T1) treated with type II‐IV resections.^48^ This suggests that on average in these patients, the tumor itself is more restrictive for dynamic range than the loss of tissue and fibrosis following the resection. The reason why the change in range SPL was larger and statistically significant in bilateral type II resections is presumably because these patients had a lower range SPL before treatment, due to the tumor on both vocal folds, resulting in worse vibration of the vocal folds preoperatively than in patients with unilateral lesions. However, improvement in range SPL may only be noticeable for the patients themselves, because changes between 3.3 and 6.6 dB as in this study are hard to distinguish for listeners. It is likely that patients will notice that less energy is needed to produce a softer or louder voice.

The mean speaking fundamental frequency increased for most patient groups. For males, the postoperative scores were all outside the range for normal values (100‐130 Hz)^24^ and was the highest in patients with a bilateral type II resection and patients with resections that involved the AC bilaterally. Voices of males having undergone unilateral type III resections, and resections with no or only unilateral involvement of the AC tended to have slightly lower postoperative fundamental frequency. For females, all mean postoperative frequencies were within the normal range (160‐230 Hz)^24^ and would not be remarkable in daily communication. The mean speaking fundamental frequency is influenced by the length, size, and tension of the vocal folds. After TLM, the vocal fold has a lower mass and is generally stiffer due to loss of the lamina propria and the introduction of fibrosis leading to higher fundamental frequency.^47^, ^48^, ^49^ Other studies have also shown an increase in fundamental frequency after treatment with TLM.^2^, ^50^, ^51^ Our results are in line with this theoretical explanation as well as with the finding in other studies. We have no explanation for the fact that the fundamental frequency decreased in two of our subpopulations above and believe this to be coincidental variation.

##### Aerodynamic parameters

The effect on aerodynamic efficiency of voicing in our study varied. In unilateral type III resections and resections not involving or with only one‐sided involvement of the AC the s/z ratio increased, moving away from the ideal value of 1. In bilateral type II resections and resections with bilateral involvement of the AC, the s/z ratio decreased and moved closer to the ideal value of 1. The preoperative and postoperative difference was significant in this latter group. This implies a higher voice efficiency was obtained after bilateral type II resections even if these lesions involved the AC. This means that after the resection, there was less air leakage or less energy was needed in order to produce vibration of the vocal folds than in patients with unilateral, though deeper, resections. Normal speaking subjects (with no deficits of the vocal folds) are able to sustain the /s/ and /z/ for about the same length of time, resulting in a s/z ratio of around 1.0. In the study by Eckel and Boone, patients with laryngeal pathology produced a mean s/z ratio of 1.4 in 95% of the time.^52^ In accordance with this study, our results showed a s/z ratio between 1.0 and 1.4 for all patients after treatment. For this aerodynamic parameter, we only found one comparable study, from Luo et al^51^ performed in Taiwan. They evaluated the long‐term voice characteristics and quality of life in 18 patients with early glottic cancer (Tis‐T2) treated with TLM. The s/z ratio that they found after treatment (1.08) is comparable with our results.^51^ Although we found one study to compare our results with, the s/z ratio is still a difficult parameter to interpret. In our study, we were not able to detect a certain pattern in this parameter.

### 4.2 Limitations

There are several limitations of this study. First, due to the longitudinal study design and the inclusion of patients in three different hospitals, we were not able to prevent the occurrence of missing data even though this was a prospective study. Therefore, we performed multiple imputation to maximize our use of the data and mitigate the problem of selective missingness. This method is an accepted and flexible method to deal with missing data, when multiple variables have missing values and observed data contains information on missing values.^53^ Second, we used the s/z ratio as an aerodynamic parameter in our study. The fact that we found no clear trend in our outcome coupled with the fact that this parameter is not commonly used in this population makes it difficult to interpret our results. We would therefore consider using a different aerodynamic parameter in further studies. Third, the subgroups regarding the ELS classification and the AC involvement were of unequal size. According to the AC involvement, 27, patients had a unilateral and 24 a bilateral resection involving the AC as opposed to 13 without. This difference in group size may have affected the outcomes of our study. This is of particular note as contrary to most studies, we did not find that involvement of the AC affected the voice outcome. However, we believe that this is mainly due to the limited size of the resections and not to a statistical flaw. Finally, a common problem in voice research is the large variety of voice tests used within the different dimensions of voice analysis and the lack of standardization of data collection, especially in objective evaluation (aerodynamic and acoustic).

## 5 CONCLUSION

On average, the voice in patients treated for early glottic carcinoma (extended T1 and limited T2) with unilateral transmuscular (type III) or bilateral subligamental (type II) resections can be expected to have mild to very moderate dysphonia 1 year postoperatively as rated by experienced listeners and by patients' self‐assessment. Dynamic range will be in the low normal to slightly decreased range and fundamental frequency will be higher than normal in male but not in female patients. Compared to preoperatively, the majority of patients can expect an improvement in their voice parameters translating to a clinically relevant improvement in voice handicap (VHI) in a third (36%) or a similar degree of voice handicap in almost half (46%) of patients 1 year postoperatively. A minority of patients (20%) will report a clinically relevant increase in voice handicap. These aforementioned findings are in line with current literature and are important to incorporate into discussion with patients during counseling for treatment options. We consider these results to be functionally acceptable and TLM to be a valid option in these patients from a functional standpoint.

## CONFLICT OF INTEREST

The authors declare that they have no conflict of interest.
